# Neonatal intestinal obstruction associated with situs inversus totalis: two case reports and a review of the literature

**DOI:** 10.1186/s13256-017-1423-z

**Published:** 2017-09-18

**Authors:** Rahul Gupta, Varsha Soni, Prakash Devidas Valse, Ram Babu Goyal, Arun Kumar Gupta, Praveen Mathur

**Affiliations:** 1 Department of Paediatric Surgery, SMS Medical College, Jaipur, Rajasthan 302004 India; 2 Department of Paediatric Surgery, JLN Medical College, Ajmer, 305001 India; 3 Department of Surgical Gastroenterology, Nizam’s Institute of Medical Sciences, Hyderabad, India

**Keywords:** Association, Jejunoileal atresia, Malrotation, Midgut volvulus, Neonatal intestinal obstruction, Situs inversus totalis

## Abstract

**Background:**

The association of neonatal intestinal obstruction with situs inversus totalis is extremely rare with only few cases reported in the literature to date. This association poses dilemmas in management. We present two such cases (of Indian origin), and briefly discuss the pertinent literature and measures to prevent unfavorable outcome.

**Case presentation:**

Case 1: a 1-month-old preterm (1300 g) male neonate belonging to Hindu (Indian) ethnicity presented with recurrent bile-stained vomiting, non-passage of stools and epigastric fullness. A babygram and upper gastrointestinal contrast studies revealed situs inversus and suggested proximal jejunal obstruction with midgut volvulus. Exploration confirmed situs inversus totalis along with reverse rotation and midgut volvulus. There was a small gangrenous area in the proximal jejunal loop. A Ladd’s procedure, resection of the gangrenous jejunal loop, and jejunojejunal anastomosis was performed. Note was made of the unusual appearance of the intestines suggestive either of fibrous or fatty infiltration. Postoperatively, our patient developed septicemia and died.

Case 2: a 4-day-old female neonate belonging to Hindu (Indian) ethnicity, small (1320 g) for gestation, presented with history of non-passage of meconium since birth, refusal to accept feeds, and episodes of recurrent bilious vomiting with abdominal distension. A plain radiogram revealed situs inversus and proximal jejunal obstruction. Ultrasonography of her abdomen revealed renal dysplastic changes in both her kidneys. Laparotomy confirmed multiple jejunoileal atresias with situs inversus totalis. Resection anastomoses was performed for multiple atresias. Our patient passed a few pellets of meconium stools postoperatively; feeds were started gradually on the sixth day. Our patient gradually developed oliguria and renal failure, followed by respiratory distress and generalized edema requiring ventilatory support. She died later due to multiorgan failure.

**Conclusions:**

Clinicians should have high index of suspicion for malrotation with midgut volvulus or intestinal atresias in neonates of situs inversus presenting with bilious vomiting. The surgical treatment should follow the same surgical principles. In situs inversus, because of transposition of viscera, midgut volvulus may occur in an anticlockwise direction, hence derotation is performed clockwise. Prognosis was poor in our series because of low birth weight, late presentation, presence of gangrenous locus in the small bowel and development of septicemia in our first case and multiorgan fibrosis/dysplasia in our second case. Early diagnosis and timely referral is paramount for favorable outcome.

## Background

Situs inversus is a rare condition (1 in 8,500 people) causing mirror image positioning of thoracic and abdominal organs [1, 2]. Situs inversus abdominus also known as situs inversus with levocardia or left-located heart is a condition with right-to-left reversal limited to the abdomen [3, 4]. In situs inversus totalis, other structural malformations are uncommon in most individuals, but they are slightly more frequent than in people with situs solitus [3, 4]. Many people with situs inversus are unacquainted of their unusual anomaly until they are evaluated medically for unrelated conditions. Situs inversus totalis complicating neonatal intestinal obstruction is very rare and presents dilemmas in management [5]. We describe two neonates with neonatal intestinal obstruction in association with situs inversus totalis, briefly discuss the pertinent literature and measures to prevent unfavorable outcome.

## Case presentation

**Fig. 1:**
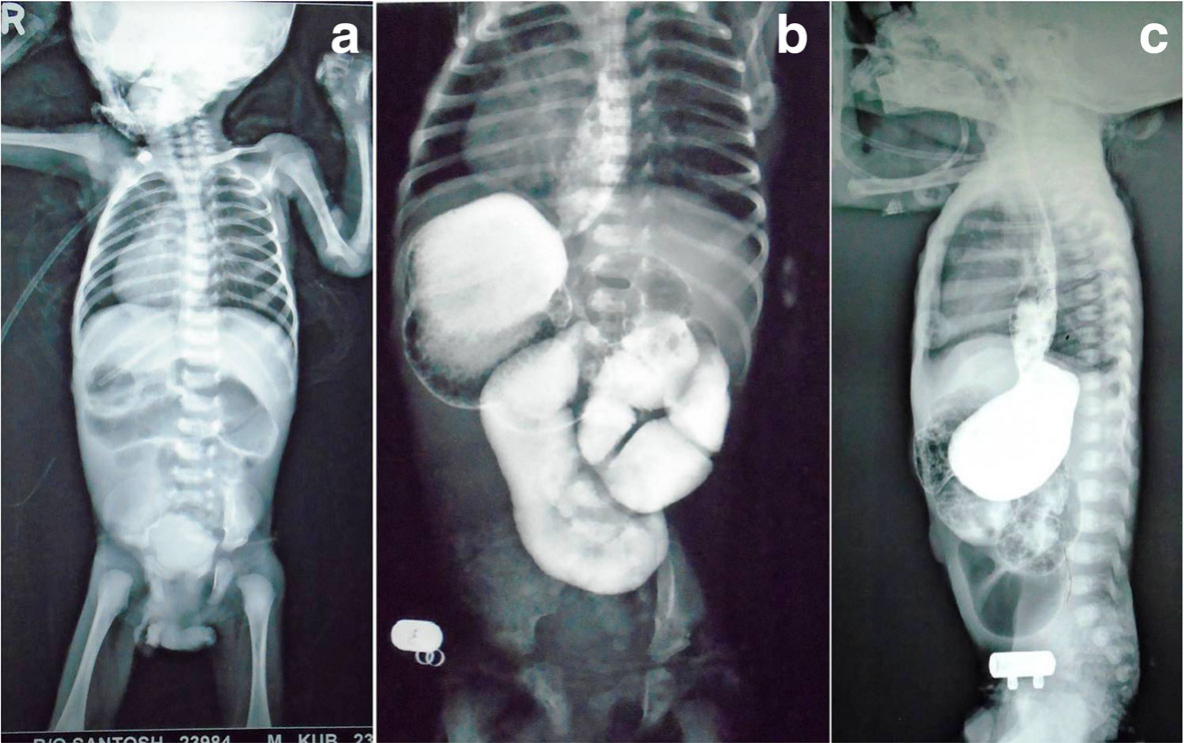
Babygram (**a**) showing dextrocardia (cardiac apex pointing to the right), liver opacity on the left side, a splenic shadow and stomach bubble on the right side of his abdomen suggestive of situs inversus. Also seen are few dilated loops in the upper abdomen with a paucity of distal gas. An upper gastrointestinal contrast study (**b** and **c**) suggestive of situs inversus, proximal jejunal obstruction, and midgut volvulus (*anteroposterior view*); gastroesophageal reflux (*lateral view*)

**Fig. 2:**
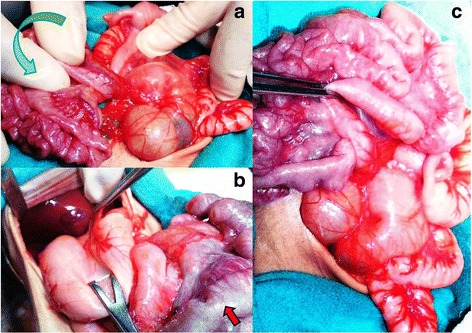
Intraoperative images showing the tip of the spleen and stomach on the right side, duodenum on the left side of his abdomen confirming situs inversus totalis. Also seen is midgut volvulus and reverse rotation (anticlockwise: *green arrow*) (**a** and **b**). Unusual appearance suggestive of either fibrous or fatty infiltration of intestines (*red arrow*) is seen. A Ladd’s procedure (**c**)

Case 1: a 1-month-old preterm, very low birth weight (1300 g), male neonate belonging to Hindu (Indian) ethnicity, presented to our hospital with recurrent bile-stained vomiting, non-passage of stools and epigastric fullness for the last 2 weeks. Antenatal ultrasounds had not been done. On examination, the neonate was afebrile, moderately dehydrated with a pulse rate of 170 beats per minute and a respiratory rate of 62 breaths per minute. Chest auscultation revealed cardiac apex on the fifth right intercostal space along the midclavicular line. The abdomen was soft and distended and nasogastric aspirate was bilious. Laboratory investigations revealed a total lymphocyte count (TLC) of 16,200 mm^3^, a hemoglobin level of 11.6 g, and raised C-reactive protein levels. A babygram revealed dextrocardia, liver opacity on the left side of his abdomen, a splenic shadow and stomach bubble on the right side, which was suggestive of situs inversus totalis. There were few dilated bowel loops in his upper abdomen with a paucity of distal gas shadows (Fig. 1a). Upper gastrointestinal (UGI) contrast studies suggested a proximal jejunal obstruction and midgut volvulus (Fig. 1b, c). Preoperative optimization was performed and consent was taken for surgical intervention. Laparotomy findings confirmed situs inversus totalis with his liver and duodenum on the left side, his stomach and spleen on the right side of his abdomen along with malrotation and midgut volvulus with reverse rotation; the caecum and appendix were present in his left upper abdomen (Fig. 2a-c). There was a small gangrenous area in the proximal jejunal loop. A Ladd’s procedure was performed along with resection of the gangrenous jejunal loop (a few centimeters) and jejunojejunal anastomosis. Note was made of the unusual appearance of his intestines suggestive of either fibrous or fatty infiltration. Postoperatively, our patient passed greenish mucoid stool on the fourth postoperative day, but later developed septicemia with a precipitous downhill course, and finally died on the sixth postoperative day.

**Fig. 3:**
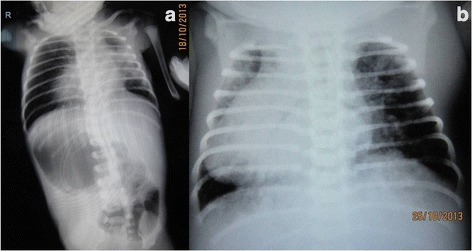
Plain roentgenograms of her chest and abdomen revealing dextrocardia, liver opacity on the left side of her abdomen, and a stomach bubble on the right side suggestive of situs inversus. Few dilated loops in upper abdomen with absence of distal air (**a**). A postoperative X-ray showing a dilated cardiac shadow on the right side along with infiltration of the lung fields (**b**)

**Fig. 4:**
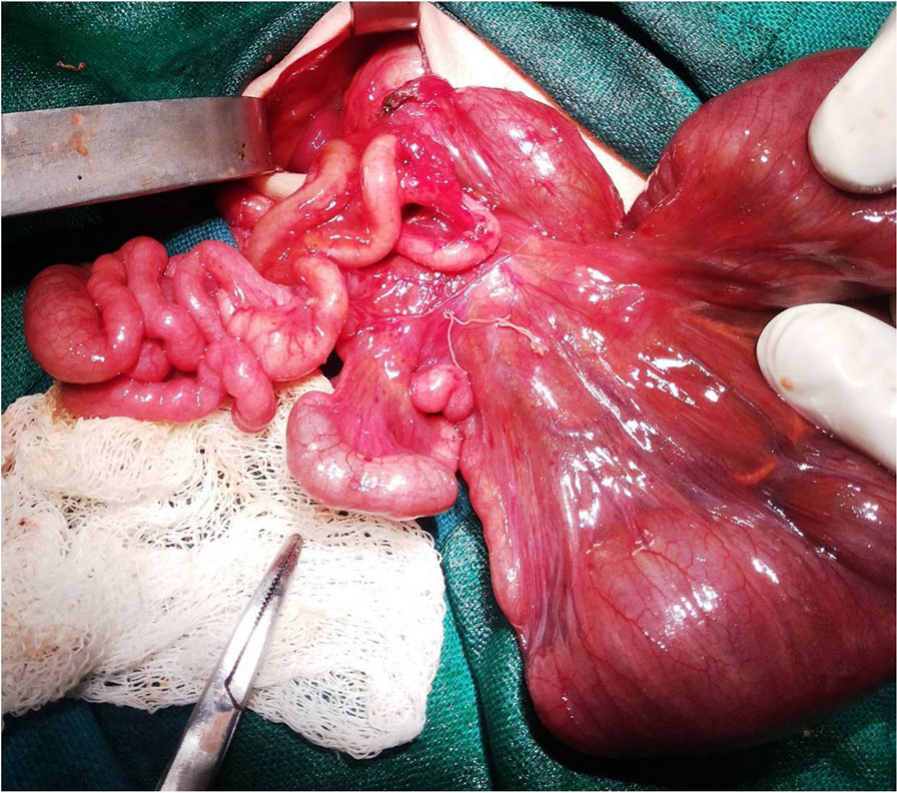
Intraoperative images revealing the tip of the spleen (*right side*), dilated jejunum, and multiple jejunoileal atresias

Case 2: a 4-day-old female neonate belonging to Hindu (Indian) ethnicity, full-term small (1320 g) for gestation, presented with non-passage of meconium since birth, refusal to accept feeds, and episodes of recurrent bilious vomiting with abdominal distension. There was no history of consanguinity. On examination, the neonate was hemodynamically stable, dehydrated, and mildly icteric with a pulse rate of 162 beats per minute and respiratory rate of 50 breaths per minute. There was upper abdominal distension with visible dilated bowel loops. Plain radiographs revealed dextrocardia, liver opacity on the left side of her abdomen, and a stomach bubble on the right side suggestive of situs inversus totalis. There were few dilated bowel loops in her upper abdomen with absence of distal gas shadows (Fig. 3a). Laboratory values revealed grossly deranged renal functions with abnormally raised urea (89 mg/dL) and creatinine levels (3.5 mg/dL), initially considered to be due to dehydration. Our patient was stabilized and resuscitated; a renal profile showed slightly improved creatinine levels (2.9 mg/dL). Ultrasonography of her abdomen revealed renal dysplastic changes in both kidneys. After optimization, an informed written consent was taken for an operative procedure. Abdominal exploration confirmed multiple jejunoileal atresias with situs inversus totalis (Fig. 4). The proximal 15 cm of the dilated jejunum was resected and multiple anastomoses with end-to-back and end-to-end anastomoses and a Heineke–Mikulicz repair were performed for multiple atresias and webs. Our patient passed a few pellets of meconium stools on the fourth postoperative day. Oral feeding was initiated on the sixth day with gradual increments. Our patient passed a few pellet-like meconium stools every second or third day. She did not develop any episode of postoperative vomiting or abdominal distension. Later, our patient redeveloped oliguria with deranged renal parameters (renal failure), respiratory distress, and generalized edema requiring ventilatory support. A chest radiograph performed on the 13^th^ postoperative day revealed infiltration of the lung fields (Fig. 3b), which was hypothesized as pneumonitis. Our patient died on the 18^th^ postoperative day due to multiorgan failure.

## Discussion

Situs inversus is a congenial anomaly with inverted position or transposition of internal organs from their normal anatomical position [1]. It is also known as situs inversus viscerum or situs transversus, or opposites [1]. The normal anatomical position of internal organs is termed as situs solitus [3]. Situs inversus was first described in humans by Fabricius, while Vehemeyer gave the radiological diagnosis [6, 7]. In situs inversus totalis, there is complete right to left reversal of all of the viscera including dextrocardia; the morphologic right atrium is on the left and the left atrium is on the right. The normal pulmonary anatomy is reversed such that the left lung has three lobes and the right lung has two. The liver and gallbladder are located on the left, and spleen and stomach are on the right side [3–7]. Associated anomalies with situs anomaly are congenital heart disease (CHD) or splenic malformations [2, 8]. Situs inversus may be asymptomatic and diagnosed incidentally during laparotomy or autopsy [8]. But, when associated with midgut volvulus or atresias, it would present early in the neonatal age, as seen in the present series [8, 9].

Heterotaxy syndrome or situs ambiguous (4 per 1 million live births) is defined as the abnormal arrangement of organs and vessels as opposed to the orderly arrangement in situs solitus or situs inversus [10]. It has three major subtypes based on the spleen: (a) asplenia (right atrial isomerism), (b) polysplenia (left atrial isomerism), (c) normal spleen located in the right upper quadrant of the abdomen. It is more commonly associated with CHD (50–90%), malrotation (70–100%) with or without midgut volvulus, and other visceral defects [10, 11].

Etiologic factors implicated in situs inversus are autosomal recessive gene, maternal diabetes, cocaine abuse, retinoic acid exposure, conjoined twinning, consanguinity and familial (multiple inheritance patterns) [1–3, 12]. There was no history of consanguinity, family history of congenital anomalies, or drug exposure in our series. There is no sex predilection, but in situs ambiguous boys predominate [13].

Situs inversus presenting in the neonatal period with gastrointestinal complaints is typically an abdominal emergency [13]. The diagnosis of situs inversus in association with neonatal intestinal obstruction is suggested by radiographs and confirmed by upper gastrointestinal contrast studies, as performed in case 1 [13]. Ultrasound and computed tomography of the abdomen may reveal the inverse relationship of superior mesenteric vessels in patients with malrotation; a whirlpool sign is seen in midgut volvulus [14]. Situs inversus represents a spectrum of rotational anomalies of gastrointestinal tract [8]. Differential diagnosis includes multiple organ malrotation syndrome [15].

Echocardiography is important to evaluate CHD. It could not be performed due to resource limitations in our series. An easy way of predicting the likelihood of CHD in a neonate with a situs abnormality (excluding situs ambiguous) is to observe the laterality of stomach and cardiac apex. If the both are on the same side of the body, the risk of associated CHD is relatively low, whereas if they are on opposite sides, the risk is relatively high. Thus, severity of CHD is proportional to the failure of cardiac shift with respect to the cardiac situs within the thorax [10]. CHD is associated with poor survival in neonates with situs abnormality.

The association of neonatal intestinal obstruction (midgut volvulus or multiple intestinal atresias) with situs inversus abdominus is rare, but its association with situs inversus totalis is extremely rare [6, 9, 16].The presence of multiple jejunoileal atresias in our second case could be due to vascular insult as a consequence of the situs anomaly. Malrotation and midgut volvulus in our first case occurred anticlockwise as compared to a clockwise rotation in classical volvulus.

Gastrointestinal anomalies associated with situs inversus are duodenal atresia, annular pancreas, biliary atresia, preduodenal portal vein, diaphragmatic hernia, lung cyst, genitourinary anomalies, ear, eye, and vertebral defects [13]. Association of situs inversus totalis with a varying spectrum of renal dysplasia, pancreatic dysplasia/fibrosis/cysts, interstitial (lung fibrosis), and intrahepatic biliary dysgenesis and meconium ileus have been reported earlier [17–20]. The second case in our series had intrauterine growth retardation, bilateral renal dysplastic changes, passed pellets of meconium stool (as seen in meconium ileus), and developed infiltration of the lung fields. Although an exact pathological diagnosis could not be established (with the parents not consenting to an autopsy), gross abnormalities indicated a diagnosis of multiorgan fibrosis/dysplasia, similar to the Japanese report [17]. Intrauterine growth retardation was also seen in cases reported by Balci *et al.* [20]. This syndrome had an unfavorable outcome in most patients [17–20]. Antenatal diagnosis of this syndrome with its known lethality (as seen in our second case) should raise the question of continuation of such pregnancy.

The treatment of neonatal intestinal obstruction associated with situs inversus totalis should follow the same surgical principles as performed for classical cases. Appropriate incision placement is required. Elective intervention is always better tolerated than an emergency surgery [11]. The classic treatment for malrotation is the Ladd procedure [21]. In situs inversus, because of transposition of viscera, the direction of rotation should be checked as midgut volvulus may occur in an anticlockwise direction, necessitating derotation in a clockwise manner (as performed in our first case) [6, 16].

In our series, unfavorable outcome in our first case was due to a combination of factors, like late presentation, low birth weight, presence of gangrenous locus in the small bowel, superimposed by septicemia. Thus, early diagnosis and timely referral along with strict asepsis is paramount for a favorable outcome. In our second case, we believe that, because of the presence of other severe anomalies, the condition was incompatible with life and further research is required to come to any final conclusion.

## Conclusions

Clinicians should have high index of suspicion for neonatal intestinal obstruction in a situs inversus patient presenting with bilious vomiting. In contrast, clinicians should look for situs anomaly when viewing plain radiographs of pediatric patients with malrotation or jejunoileal atresias. The surgical treatment should follow the same surgical principles as performed for classical cases. In situs inversus, because of the transposition of viscera, midgut volvulus may occur in an anticlockwise direction, hence derotation is performed clockwise. Prognosis was poor in our series because of low birth weight, late presentation, presence of gangrenous locus in the small bowel, and development of septicemia in our first case and multiorgan fibrosis/dysplasia in our second case. Early diagnosis and timely referral is paramount for a favorable outcome.
